# Delayed Toxic-Hypoxic Leukoencephalopathy As Sequela of Opioid Overdose and Cerebral Hypoxia-Ischemia

**DOI:** 10.7759/cureus.20271

**Published:** 2021-12-08

**Authors:** David Chachkhiani, Anil K Chimakurthy, Olinda Verdecie, Cheryl T Goyne, Edward C Mader

**Affiliations:** 1 Neurology, Louisiana State University Health Sciences Center, New Orleans, USA

**Keywords:** myelinopathy, eeg, demyelinating, opioid, ischemia, hypoxia, toxic, delayed, leukoencephalopathy

## Abstract

Delayed leukoencephalopathy in the aftermath of toxic exposure and cerebral hypoxia-ischemia is known as “delayed post-hypoxic leukoencephalopathy” (DPHL) but the name “delayed toxic-hypoxic leukoencephalopathy” (DTHL) may be more accurate if toxic and hypoxic mechanisms are both involved in the pathogenesis of delayed leukoencephalopathy. DTHL is characterized by initial recovery from toxic exposure and cerebral hypoxia-ischemia, clinical stability over a few weeks, and subsequent neurological deterioration with the sudden emergence of diffuse white matter disease. A 46-year-old man suffered respiratory failure and hypotension as a result of opioid overdose. Brain MRI showed watershed infarcts and EEG showed diffuse theta-delta slowing consistent with global cerebral hypoperfusion. He recovered fully and was discharged with intact cognitive function. Three weeks later, he presented with abulia and psychomotor retardation. MRI revealed extensive white matter hyperintensity and EEG showed diffuse polymorphic delta activity. DTHL was diagnosed based on classic MRI features, history of opioid overdose and hypoxic brain injury, and negative test results for etiology of white matter disease. He developed akinetic mutism prompting administration of methylprednisolone 1000-mg IV q24h for five days. He also received amantadine 100-mg PO q12h. His cognition, motivation, and psychomotor function slowly improved and returned to baseline about two months after the overdose. Clinic reassessment two and a half months after the overdose revealed normal cognitive function, slight residual MRI hyperintensity, and mild EEG slowing anteriorly. Toxic-metabolic myelinopathy causing diffuse demyelination in the deep white matter is a perfect explanation for the patient’s neurological symptoms, MRI changes, EEG findings, and time course of recovery.

## Introduction

Delayed post-hypoxic leukoencephalopathy (DPHL), also known as Grinker myelinopathy, delayed anoxic encephalopathy, or post-interval syndrome, is a rare complication of hypoxic-ischemic brain injury [1]. DPHL is characterized by a biphasic clinical course, with initial complete or near-complete recovery from cerebral anoxia, followed by clinical stability over a period of weeks (lucid interval), and subsequent neurological deterioration due to diffuse white matter disease [2]. It has nearly been a century since Grinker recognized the link between delayed leukoencephalopathy and anoxic brain injury [3]. In 1962, Plum et al. reported cases of delayed encephalopathy outside the context of intoxication, e.g., after cardiac arrest or surgery complicated by cerebral hypoperfusion [4]. In the vast majority of DPHL cases, the hypoxic event can be traced to a neurotoxic event, notably carbon monoxide poisoning or drug overdose [1,2].

Carbon monoxide poisoning was the cause of anoxic brain injury in the initial reports of DPHL [4-6]. Ginsberg retrospectively studied nine patients with delayed postanoxic encephalopathy after carbon monoxide poisoning: three had a full recovery, two survived but had permanent neurologic sequelae and personality changes, and four died [5]. In the cohort of Choi et al., patients who suffered carbon monoxide poisoning went through a lucid interval that lasted between two and 40 days (average was 22 days) before showing signs of delayed neurological deterioration [6]. DPHL has been increasingly linked to drug overdose in recent years [7-9]. It is possible that opioid overdose has already overtaken carbon monoxide poisoning as the leading cause of DPHL.

The notion that delayed leukoencephalopathy is an aftermath of cerebral hypoxia-ischemia encouraged the use of the term “DPHL” in the literature. Munoz et al. recently reported three cases of delayed leukoencephalopathy following opioid-induced anoxic coma and pointed out that toxic drug exposure may have been overlooked in previous case reports of DPHL [9]. The authors adapted the less committal term “delayed toxic-hypoxic leukoencephalopathy” (DTHL) to emphasize the interaction between toxin-induced and hypoxia-induced mechanisms in the pathogenesis of delayed leukoencephalopathy [9]. We describe a case of DTHL complicating opioid overdose and cerebral hypoxia-ischemia with neurological, MRI, and EEG manifestations that could be explained best by demyelination in the deep white matter due to toxic-metabolic myelinopathy.

## Case presentation

**Figure 1:**
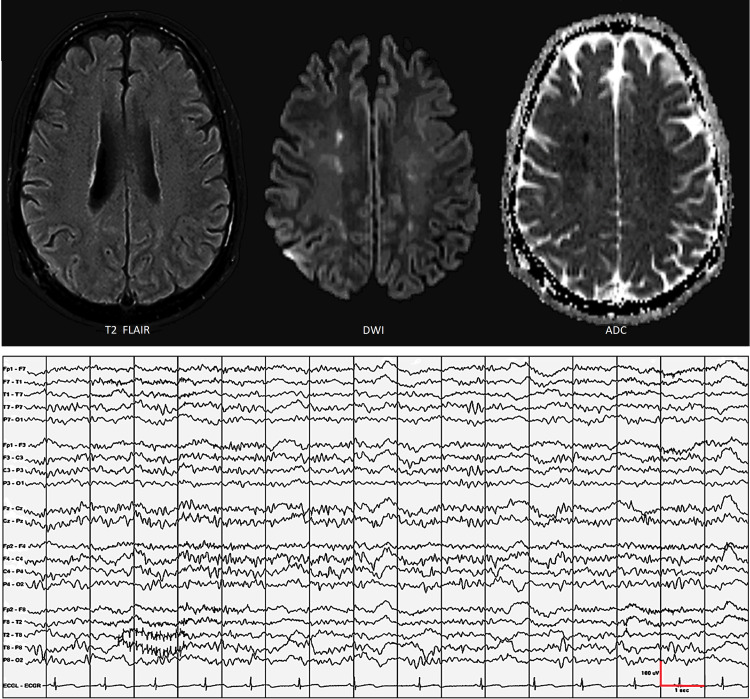
During initial admission because of acute opioid overdose, complicated by acute respiratory failure, hypotension, and obtundation, brain MRI showed watershed infarcts in the centrum semiovale and EEG showed diffuse theta-delta activity consistent with global hypoperfusion.

A 46-year-old Caucasian male, with a history of substance abuse, hepatitis C, and mesial temporal lobe epilepsy, presented with abulia and psychomotor retardation. Twenty-seven days earlier (this is our reference point or day 1), he was admitted to another hospital because of acute opioid overdose (confirmed by qualitative and quantitative toxicology). Emergency responders found him comatose (GCS=4, E1V1M4) in sinus tachycardia (HR = 132/min) with agonal respiration (RR = 10/min). En route to the hospital, 100% oxygen was administered and naloxone 2-mg IV was injected. On arrival, respiratory rate increased to 18/min but he remained comatose (GCS=4, E1V1M4). Blood oxygen saturation (on 100% oxygen) dropped from 94% to 85% and blood pressure dropped from 98/60 mmHg to 80/42 mmHg. At that point, he was intubated, mechanical ventilation was started, and he was admitted to the intensive care unit. He only suffered respiratory failure and hypotension, not cardiac arrest. Vital signs and oxygen saturation were stabilized approximately 25 minutes after initial contact with emergency responders. Brain MRI showed watershed infarcts in the centrum semiovale and EEG showed diffuse theta-delta activity consistent with global hypoperfusion (Figure 1). He was stepped down after five days and he was discharged after eight days with normal cognitive and neurological function. At home, he was able to engage in normal activities of daily living for 19 days before he developed acute-onset abulia and psychomotor retardation.

**Figure 2:**
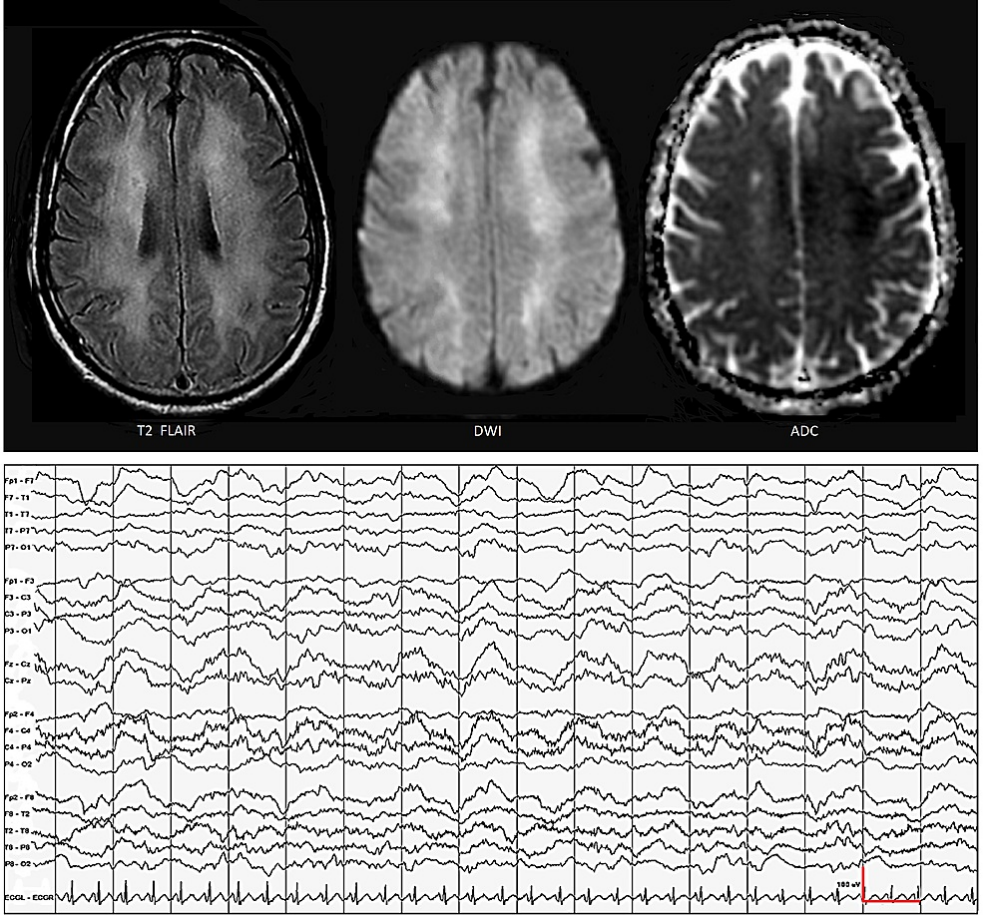
During readmission for abulia and psychomotor retardation, brain MRI showed extensive bilaterally symmetric deep white matter hyperintensity with patchy areas of restricted diffusion and EEG showed diffuse polymorphic delta activity indicating widespread demyelination or axonal injury in the white matter.

The patient was taken to the emergency room and readmitted on day 27. On examination, he was awake and oriented, but inattentive and apathetic, with a paucity of spontaneous movements. His responses were delayed and limited to one or two words. Pulse rate was 96/min, respiratory rate 16/min, blood pressure 112/68 mmHg, and temperature 36.5 ºC. He had diffuse hyperreflexia but other neurological deficits were absent. On day 28, brain MRI showed symmetric diffuse white matter hyperintensity with patchy areas of restricted diffusion and EEG showed diffuse polymorphic delta activity (Figure 2). All blood studies were normal. Toxicology was positive only for phenobarbital, a drug prescribed by his primary care physician for epilepsy. Antigen/antibody testing showed that he was positive for hepatitis C virus (HCV) and negative for hepatitis A virus (HAV), hepatitis B virus (HBV), and HIV. Blood tests were also negative for autoimmune markers, including erythrocyte sedimentation rate (ESR), rapid plasma reagin (RPR), antinuclear antibody test (ANA), rheumatoid factor, C3/C4 complement, and cryoglobulins. Cerebrospinal fluid (CSF) studies showed elevated protein (108 mg/dL) but normal cell count (WBC <5/cu mm, no RBC) and glucose level (59 mg/dL). CSF was negative for John Cunningham (JC) virus, herpes simplex virus type 1 (HSV1), herpes simplex virus type 2 (HSV2), and arboviruses. CSF was also negative for myelin basic protein, oligoclonal bands, and anti-neuronal antibodies. DTHL was diagnosed based on the history of cerebral hypoxia-ischemia, the 19-day lucid interval, and the absence of an alternative explanation for the white matter disease.

**Figure 3:**
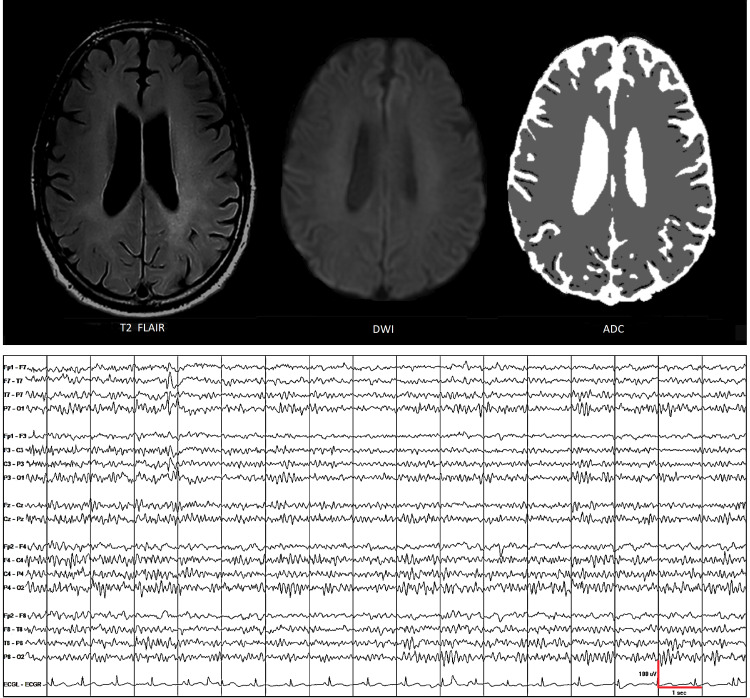
During his clinic follow-up, approximately two and a half months after the overdose, brain MRI showed near-complete resolution of white matter hyperintensity and EEG showed irregular theta and delta waves anteriorly and normal alpha rhythm posteriorly. Epileptiform sharp waves occurred infrequently over the left temporal head region, but this finding is not new (the patient has mesial temporal lobe epilepsy).

Abulia and psychomotor retardation progressed to akinetic mutism on day 32 prompting initiation of methylprednisolone 1000-mg IV q24h. He completed five days of steroid therapy. Verbal response and psychomotor function started to improve. On day 38, he was transferred to the long-term acute care section of the hospital. Amantadine 100 mg q12h was also started. He underwent physical therapy, occupational therapy, and speech therapy. His verbal ability and psychomotor function showed further improvement. He only had mild abulia when he was discharged on day 48. He continued taking amantadine at home and was essentially back to his normal baseline on day 62, approximately two months after the overdose. During his clinic follow-up on day 138, approximately two and a half months after the overdose, his cognitive and neurological functions were normal (except for diffuse hyperreflexia), brain MRI showed near-complete resolution of white matter hyperintensity, and EEG showed irregular theta and delta waves anteriorly and normal alpha rhythm posteriorly (Figure 3).

## Discussion

The cause of delayed white matter deterioration in DTHL is unknown [10]. A double-hit mechanism was hypothesized whereby toxic-induced and post-hypoxic processes interact and give rise to metabolic dysregulation and myelinotoxicity [7]. Whatever pathogenetic mechanism is proposed must necessarily explain the following facets of DTHL: (1) its rarity, even though drug overdose and cerebral hypoxia-ischemia are relatively common; (2) its delayed onset, with two to five weeks of lucid interval following the hypoxic event; (3) its reversibility and time course of recovery; 4) its MRI manifestation as diffuse non-enhancing white matter disease with scattered areas of restricted diffusion; and (5) its neuropathological characteristics, notably demyelination with preservation of axons and oligodendrocytes.

Drug overdose and cerebral hypoxia-ischemia are common, but DTHL is rare, indicating that DTHL requires another precondition, such as genetic susceptibility. A partial deficit of aryl-sulfatase A, a key enzyme in myelin metabolism, was reported in DTHL [11,12] but this finding was not consistently replicated [1,13]. The delayed onset of DTHL after the hypoxic event (lucid interval) can be explained by alteration in myelin turnover due to oligodendrocyte dysfunction [9,10]. The half-life of the fast component of the myelin basic protein pool (19 to 22 days) matches the duration of the lucid interval [12,14]. Evidently, impairment of oligodendrocytes myelin protein synthesis by the initial toxic-hypoxic event results in preserved function during the lucid interval followed by acute functional decompensation due to deficient or abnormal myelin replacement [1,10]. The lucid interval can also be explained on the basis of delayed oligodendrocyte apoptosis or delayed cell-mediated autoimmunity [15] but both of these mechanisms would not explain some of the clinical, MRI, and histopathological features of DTHL.

Brain MRI showed changes typical of DTHL in our patient. FLAIR and DWI sequences revealed diffuse hyperintensity in the centrum semiovale with sparing of the U-fibers, corpus callosum, brainstem, and cerebellum. Patchy areas of restricted diffusion were also noted in the centrum semiovale. Gray matter restricted diffusion is frequently attributed to cytotoxic edema and white matter restricted diffusion to axonal swelling. However, white matter restricted diffusion can also be explained on the basis of intramyelinic edema or water trapping in demyelinated or myelinopathic areas [1,16]. The MRI findings in our patient are in agreement with published pathology reports in DTHL (biopsy was not performed on our patient). Histopathologically, DTHL is characterized by patchy areas of demyelination in the deep white matter with reactive astrocytosis and relative preservation of axons and oligodendrocytes [3,4,13]. There is absent or minimal interstitial edema, lymphocytic infiltration, and microgliosis. Lesions in the globus pallidus or neocortex have been detected in DTHL, but these lesions are most likely caused by the hypoxic event preceding DTHL [5-9].

EEG is often not included in the workup of acute diffuse leukoencephalopathy. This is unfortunate since EEG abnormalities can suggest the dominant mechanism of white matter disease. No EEG change or minimal slowing suggests white matter edema while diffuse polymorphic delta activity (as in our patient’s EEG) indicates diffuse axonal injury or extensive demyelination [17]. The time course of clinical symptoms and abnormal MRI and EEG findings imply that demyelination, not axonal loss, is the fundamental mechanism of DTHL in our patient. The MRI changes in DTHL are reversible with follow-up MRI showing resolution or significant reduction in white matter hyperintensity when the patient’s cognitive and neurological function is back to baseline. Taken together, these findings point towards deep white matter demyelination as the pathological basis of DTHL.

DTHL is remarkably different from multiple sclerosis, osmotic demyelination syndrome, and progressive multifocal leukoencephalopathy in terms of clinical, radiological, and histopathological characteristics suggesting that the pathogenesis of demyelination in DTHL is fundamentally different from these more common demyelinating diseases of the brain [15]. The most plausible mechanism of demyelination in DTHL is toxic-metabolic myelinopathy [9]. Carbon monoxide is certainly myelinotoxic, but opioids and other drugs can also be myelinotoxic if ingested in large amounts, especially if complicated by cerebral hypoxia-ischemia. Toxic exposure may cause myelinopathy and subsequent demyelination by impairing ATP-dependent enzymes responsible for myelin turnover in the deep white matter [1,15]. Deep white matter injury in DTHL may be a manifestation of the vulnerability of the deep white matter to hypoxia-ischemia [2]. Unlike the corpus callosum and U-fibers, the deep white matter is supplied by widely spaced arterioles with few anastomoses [1,18]. Indeed, our patient’s MRI revealed watershed infarcts in the centrum semiovale as a result of respiratory failure and hypotension caused by opioid overdose.

There is limited data on the effective treatment of DTHL. We are not sure if the five-day course of methylprednisolone reduced the severity of DTHL in our patient. There is a recent report of a patient with DTHL who was treated with high-dose steroids and plasmapheresis but the patient continued to deteriorate and eventually passed away [19]. We maintained our patient on amantadine even if there is only anecdotal evidence of cognitive and neurobehavioral improvement with this drug in DTHL [20]. Even if specific treatment is lacking in DTHL, complete or near-complete recovery is the rule with proper supportive care and prevention of complications. The only caveat is that DTHL must not be confused with another white matter disease.

## Conclusions

DTHL is important to recognize because failure to do so can lead to unnecessary treatments and investigations. Clinical picture, natural history, and outcome are predictable in DTHL caused by opioid overdose. In a person with a history of drug overdose, cerebral hypoxia-ischemia, and a lucid interval, MRI findings of diffuse white matter hyperintensity with areas of restricted diffusion strongly suggest DTHL. Diffuse polymorphic delta activity on EEG provides additional evidence in favor of a demyelinating process involving the deep white matter. Therefore, EEG should always be included in the work-up of acute diffuse leukoencephalopathy. Diffuse toxic-metabolic myelinopathy resulting in deep white matter demyelination is the most plausible pathogenetic mechanism in DTHL. Further investigation is needed to understand how toxic-metabolic myelinopathy differs from the more malignant forms of demyelination, such as multiple sclerosis, progressive multifocal leukoencephalopathy, and osmotic demyelination syndrome.
